# Systolic anterior motion of the anterior mitral valve leaflet begins in subclinical hypertrophic cardiomyopathy

**DOI:** 10.1093/ehjci/jead186

**Published:** 2023-07-31

**Authors:** Samuel Seitler, Surani De Zoysa Anthony, Chinwe C C Obianyo, Petros Syrris, Vimal Patel, Daniel M Sado, Viviana Maestrini, Silvia Castelletti, Stephen Walsh, Ben O’Brien, James C Moon, Gabriella Captur

**Affiliations:** UCL Institute of Experimental Medicine, Royal Free London, Gower Street, London, UK; University College London, Institute of Cardiovascular Science, Gower Street, London WC1E 6BT, UK; University College London, Institute of Cardiovascular Science, Gower Street, London WC1E 6BT, UK; NIHR University College London Hospitals Biomedical Research Center, London, UK; Barts Heart Center, The Cardiovascular Magnetic Resonance Imaging Unit and The Inherited Cardiovascular Diseases Unit, St Bartholomew’s Hospital, West Smithfield, London, UK; University College London, Institute of Cardiovascular Science, Gower Street, London WC1E 6BT, UK; University College London, Institute of Cardiovascular Science, Gower Street, London WC1E 6BT, UK; Cardiovascular Magnetic Resonance Unit, King’s College London, UK; University College London, Institute of Cardiovascular Science, Gower Street, London WC1E 6BT, UK; University College London, Institute of Cardiovascular Science, Gower Street, London WC1E 6BT, UK; Department of Nephrology, Royal Free London NHS Foundation Trust, Pond Street, London, UK; UCL Institute of Experimental Medicine, Royal Free London, Gower Street, London, UK; Department of Perioperative Medicine, St Bartholomew’s Hospital, Barts Health NHS Trust, West Smithfield, London EC1A 7BE, UK; Department of Cardiac Anesthesiology and Intensive Care Medicine, German Heart Center, Augustenburger Platz 1, 13353 Berlin, Germany; Department of Cardiac Anesthesiology and Intensive Care Medicine, Charite Berlin, Augustenburger Platz 1, 13353 Berlin, Germany; Outcomes Research Consortium, Department of Outcomes Research, The Cleveland Clinic, 9500 Euclid Ave. P77, Cleveland, OH 44195, USA; University College London, Institute of Cardiovascular Science, Gower Street, London WC1E 6BT, UK; NIHR University College London Hospitals Biomedical Research Center, London, UK; Barts Heart Center, The Cardiovascular Magnetic Resonance Imaging Unit and The Inherited Cardiovascular Diseases Unit, St Bartholomew’s Hospital, West Smithfield, London, UK; University College London, Institute of Cardiovascular Science, Gower Street, London WC1E 6BT, UK; MRC Unit of Lifelong Health and Ageing, 1 – 19 Torrington Place, London WC1E 7HB, UK; Department of Cardiology, Royal Free Hospital NHS Foundation Trust, Pond Street, Hampstead, London NW3 2QG, UK

**Keywords:** hypertrophic cardiomyopathy, mitral valve, anterior mitral valve leaflet

## Abstract

**Aims:**

Anterior mitral valve leaflet (AMVL) elongation is detectable in overt and subclinical hypertrophic cardiomyopathy (HCM). We sought to investigate the dynamic motion of the aorto-mitral apparatus to understand the behaviour of the AMVL and the mechanisms of left ventricular outflow tract obstruction (LVOTO) predisposition in HCM.

**Methods and results:**

Cardiovascular magnetic resonance imaging using a 1.5 Tesla scanner was performed on 36 HCM sarcomere gene mutation carriers without left ventricular hypertrophy (G+LVH−), 31 HCM patients with preserved ejection fraction carrying a pathogenic sarcomere gene mutation (G+LVH+), and 53 age-, sex-, and body surface area–matched healthy volunteers. Dynamic excursion of the aorto-mitral apparatus was assessed semi-automatically on breath-held three-chamber cine steady-state free precession images. Four pre-defined regions of interest (ROIs) were tracked: ROI_PMVL_: hinge point of the posterior mitral valve leaflet; ROI_TRIG_: intertrigonal mitral annulus; ROI_AMVL_: AMVL tip; and ROI_AAO_: anterior aortic annulus. Compared with controls, normalized two-dimensional displacement-vs.-time plots in G+LVH− revealed subtle but significant systolic anterior motion (SAM) of the AMVL (*P* < 0.0001) and reduced longitudinal excursion of ROI_AAO_ (*P* = 0.014) and ROI_PMVL_ (*P* = 0.048). In overt and subclinical HCM, excursion of the ROI_TRIG/AMVL/PMVL_ was positively associated with the burden of left ventricular fibrosis (*P* < 0.028). As expected, SAM was observed in G+LVH+ together with reduced longitudinal excursion of ROI_TRIG_ (*P* = 0.049) and ROI_AAO_ (*P* = 0.008).

**Conclusion:**

Dyskinesia of the aorto-mitral apparatus, including SAM of the elongated AMVL, is detectable in subclinical HCM before the development of LVH or left atrial enlargement. These data have the potential to improve our understanding of early phenotype development and LVOTO predisposition in HCM.

## Graphical Abstract

**Graphical Abstract:**
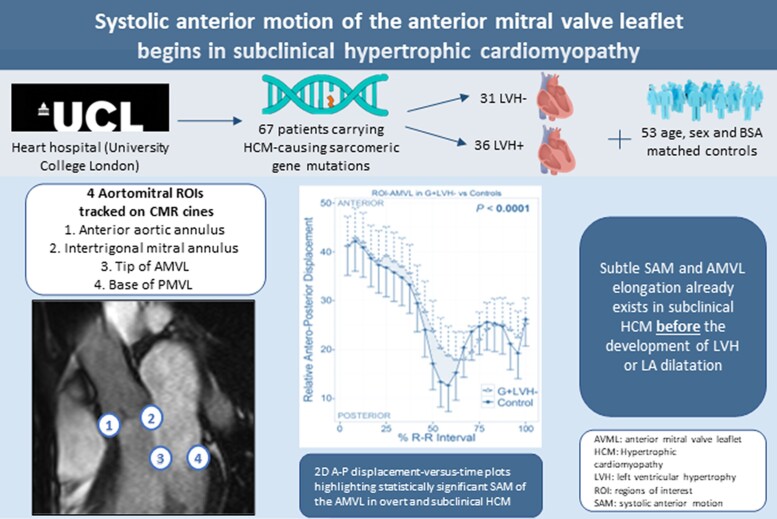
CMR cines were used to track ROIs related to the aorto-mitral apparatus to understand the mechanisms of SAM in subclinical and overt HCM. BSA, body surface area; LA, left atrium. Other abbreviations as in *Figures 1*, *2*, and *4*.

## Introduction

Mitral valve (MV) abnormalities, in particular, elongation of the anterior MV leaflet (AMVL), occur in patients with overt hypertrophic cardiomyopathy (HCM).^1^ AMVL elongation is also a recognized feature of subclinical HCM,^1,2^ that is, HCM sarcomere gene mutation carriers before the development of left ventricular hypertrophy (LVH) or left atrial (LA) enlargement (G+LVH−).

The combination of AMVL elongation and its systolic anterior motion (SAM)^3^ contributes towards pathological left ventricular outflow tract obstruction (LVOTO) and mitral regurgitation (MR) in patients with HCM expressing LVH.^4^ SAM can result in a spectrum of obstruction ranging from a clinically silent manifestation to severe haemodynamically compromising LVOTO. When evaluated carefully, LVOTO can be observed in ≈70% of patients with HCM, the vast majority of whom also expressed SAM of the MV. This is clinically significant because it has been previously demonstrated that LVOTO is independently associated with severe adverse HCM-related outcomes,^5^ including an increased risk of sudden cardiac death.^6^

Early identification of SAM and other geometric determinants of LVOTO predisposition will enhance our understanding of initial HCM phenotypic presentation and allow for timely risk stratification. Additionally, alleviation of these abnormalities early on has the potential to avert LVOTO development in G+LVH− patients and ultimately improve outcomes for this cohort. Disease-modifying therapies evidenced to improve early left ventricular (LV) remodelling in subclinical HCM are also being investigated^7^ and represent a promising therapeutic approach for these patients.

Cardiovascular magnetic resonance (CMR) imaging detects AMVL elongation in overt and subclinical HCM patients, but the kinetic and geometric interactions of the MV, subvalvular apparatus, and LV outflow tract (LVOT) are more complex to appraise. The purpose of this study was to investigate the dynamic motion of the whole aorto-mitral apparatus using a target tracking technique applied to CMR cine data to better understand the behaviour of the AMVL and provide insights into the mechanisms of LVOTO predisposition in HCM.

## Methods

### Study population

This prospective case–control study population has been previously described in detail.^8,9^ Briefly, G+LVH− and G+LVH+ HCM participants recruited from the Heart Hospital (University College London, UK) were matched to healthy volunteers on the basis of age (±8 years), sex, body surface area (BSA, ±10%), and ethnicity. Inclusion criteria for G+LVH− (*n* = 36) included the following: (i) maximal LV wall thickness < 13 mm by CMR and mass within the normal range relative to BSA, age, and sex; (ii) sinus rhythm, no LVH, and no pathological Q waves/T-wave inversion on 12-lead electrocardiography (ECG); and (iii) no causes of secondary LVH. G+LVH+ participants (*n* = 31) had a non-dilated, hypertrophied LV (maximum wall thickness ≥ 15 mm) in the absence of another cardiac or systemic disease that could produce the same magnitude of hypertrophy.^10^ Healthy volunteers (total *n* = 53) had no history of cardiovascular disease or hypertension, a normal physical examination, no family history of inheritable cardiomyopathy or sudden cardiac death, no personal history of cardiac symptoms, and a normal ECG. The control group was divided into two subgroups, matched to LVH− and LVH+ patients, with 14 subjects acting as controls for both subgroups. Exclusion criteria for all participants were the presence of conventional contraindications for CMR, claustrophobia, and arrhythmias. An ethics committee of the UK National Research Ethics Service approved the generic analysis of anonymized clinical scans. The genotyping project was approved by the UCL/UCLH Joint Research Ethics Committee. At the time of enrolment, all participants gave written informed consent conforming to the Declaration of Helsinki (V. revision, 2000).

### ECG

Standard 12-lead ECG was performed in the supine position during quiet respiration. LVH was evaluated with the Romhilt–Estes^11^ and electrocardiographic European criteria.^12,13^ ECGs were analysed by an experienced observer blinded to clinical information.

### Genetic screening

Genomic data pertaining to this cohort have been previously published.^8^ G+ individuals carried a known disease-causing mutation in one of the following sarcomere genes: myosin-binding protein C (*MYBPC3*), β-myosin heavy chain (*MYH7*), troponin T (*TNNT2*), troponin I (*TNNI3*), myosin regulatory light chain (*MYL2*), myosin essential light chain (*MYL3*), tropomyosin (*TPM1*), and cardiac α-actin (*ACTC1*).

### Transthoracic echocardiography

Resting transthoracic echocardiography was performed using Vivid i7 (General Electric Vingmed Ultrasound, Horten, Norway) and Philips Sonos 7500 (Philips Medical Systems, Andover, MA, USA) platforms within 3 months of CMR, using standard acquisition protocols.^14^ SAM was defined as incomplete if there was any movement of the MV leaflets (MVLs) or chordae towards the ventricular septal endocardium without septal contact and complete when there was contact with the ventricular septum during systole. The maximal LVOT gradient was measured using continuous wave Doppler in the apical five-chamber view at rest. Care was taken to exclude Doppler signals from any coexistent MR or mid-cavity obstruction. Resting LVOTO was defined as a peak LVOT gradient of ≥30 mmHg.

### CMR imaging

Standard clinical scans (localizers, black and white blood images, three long-axis views, and LV short-axis stack) were performed using a 1.5 Tesla magnet (Avanto, Siemens Medical Solutions®, Erlangen, Germany). CMR short-axis volumetric studies^15^ were acquired from retrospectively gated, breath-held, balanced, steady-state free precession (SSFP) cines (slice thickness, 7 mm; interslice gap, 3 mm; flip angle, 60–80°; repetition time, 3.0 ms; echo time, 1.33 ms; field of view read typically, 380 mm; phase resolution, 75%; typical acquired voxel size, 1.5 × 1.7 mm; and 12 lines per segment). Late gadolinium enhancement (LGE) images were acquired as previously described.^8^ SAM was defined as incomplete if there was any movement of the MVLs or chordae towards the ventricular septal endocardium without septal contact and complete when there was contact with the ventricular septum during systole.

### Image processing

**Figure 1:**
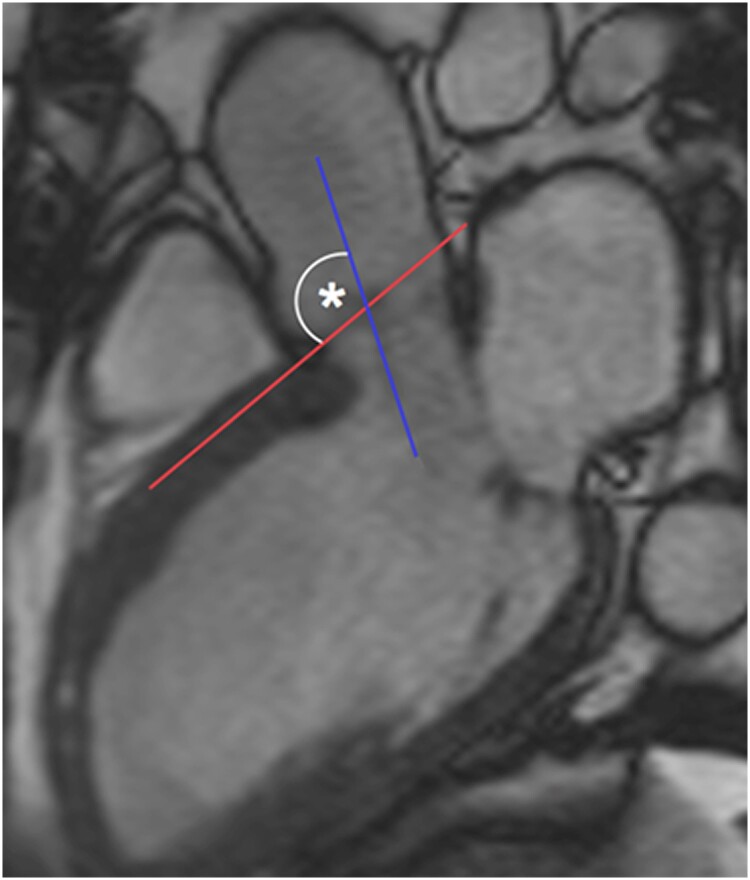
LVOT–basal septal angle measurement by CMR. The basal septal line (red) is drawn along the interventricular septum, parallel to the proximal right endocardial border. The LVOT line (blue) is drawn through the long axis of the LVOT, and the angle (*) it subtends with the septal line was measured (smaller angles represent more angulation). CMR, cardiovascular magnetic resonance; LVOT, left ventricular outflow tract.

Standard analysis of echocardiographic data was performed using EchoPAC (General Electric). CMR volumetric data were analysed in CMR tools (v.1.0, 2010). LVOT–basal septal angles were measured from three-chamber CMR cines using OsiriX® (v.6.5.2, 64-bit; *Figure 1*). Total LGE volume was quantified using the signal threshold vs. reference mean (STRM) semi-automated technique with an STRM-based threshold of >3 standard deviations (SD) above the mean signal intensity of the reference myocardium as previously described.^16^

**Figure 2:**
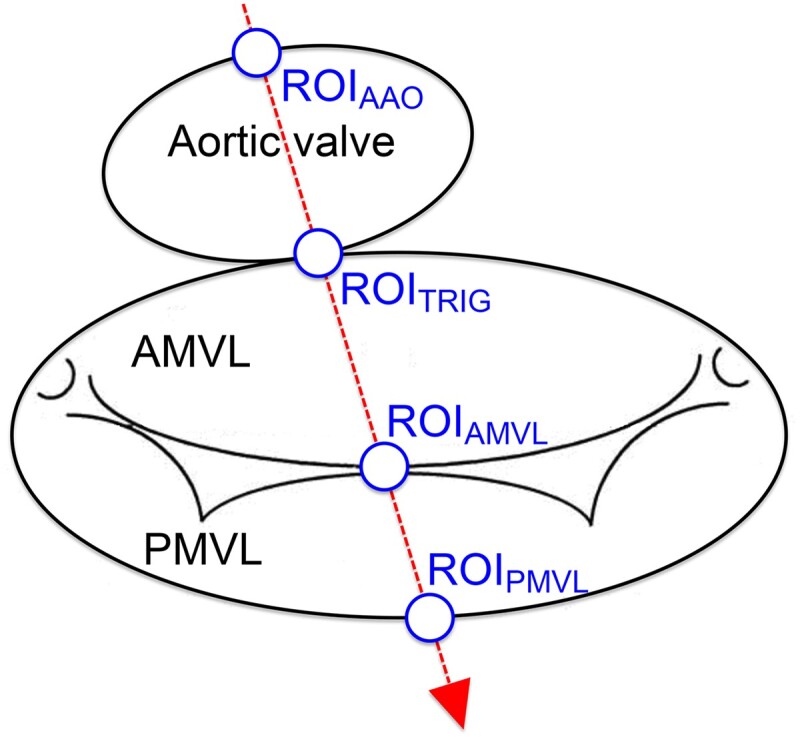
Schematic of the aorto-mitral apparatus and ROIs. The four ROIs are transected by the single plane of the three-chamber CMR view (red arrow). AMVL, anterior mitral valve leaflet; ROI, region of interest; ROI_AAO_, ROI anterior aortic annulus; ROI_AMVL_, ROI AMVL tip; ROI_PMVL_, ROI hinge point of the posterior MVL; ROI_TRIG_, ROI intertrigonal mitral annulus.

**Figure 3:**
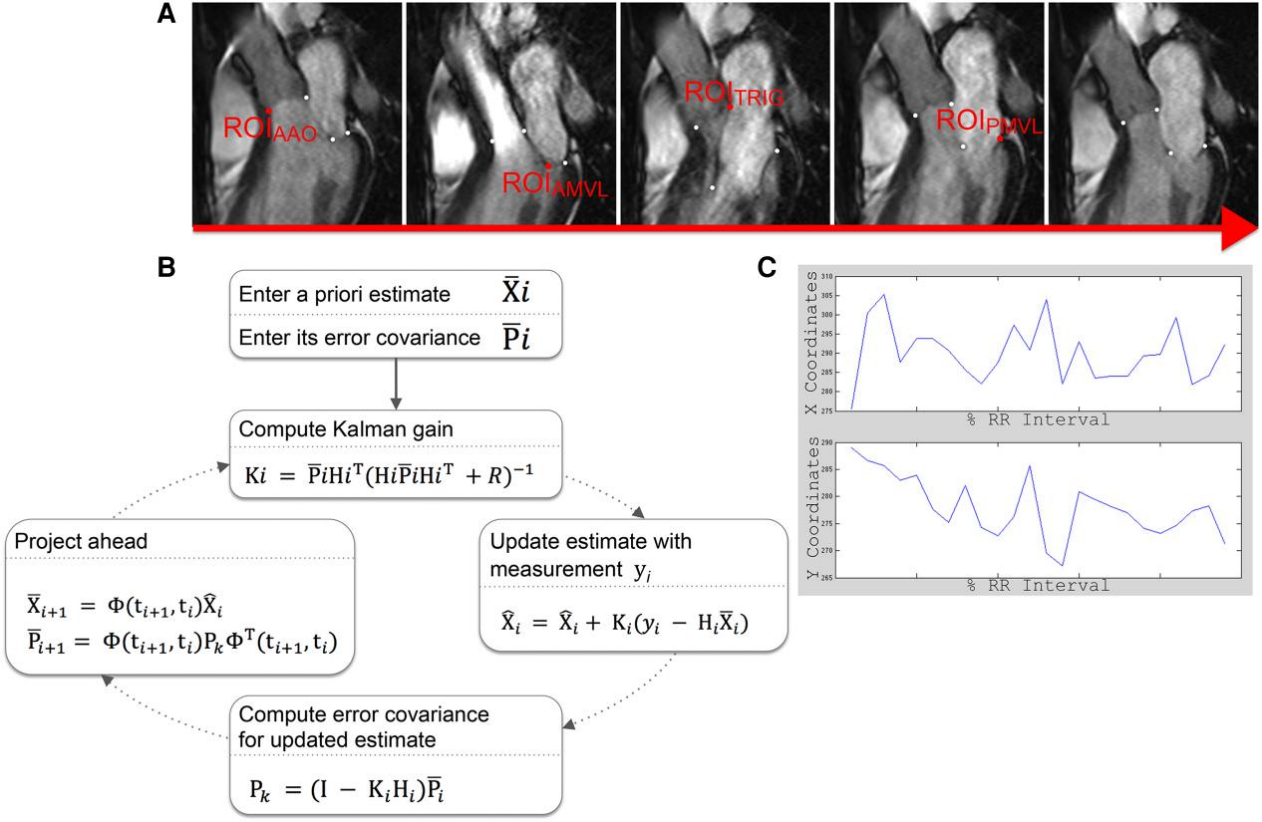
Image processing sequence for feature tracking of the aorto-mitral apparatus ROIs. (*A*) Five exemplar frames out of a typical 24-frame three-chamber cine showing the four ROIs. Each ROI is separately tracked by a modified Kalman filter algorithm that handles CMR cine images (*B*) in MATLAB, from which the *X*/*Y* coordinates summarizing its path over 1 R–R interval (*C*) are then derived. The motion tracking algorithm operated on the three-chamber views and provided motion tracking coordinates in the two-dimensional space (*X* and *Y*, throughout the cardiac cycle). In these plots, all *X*/*Y* coordinate points were normalized to allow comparison across cohorts, and they are thus plotted on the *X* and *Y* axes accordingly. Abbreviations as in *Figures 1* and *2*.

For target tracking, four pre-defined aorto-mitral regions of interest (ROIs) were labelled with white circular objects in ImageJ (1.46c, 64-bit) throughout one cardiac cycle using the three-chamber cine composed of the standard 24 frames. The four aorto-mitral ROIs (*Figure 2*) were as follows: (i) ROI_PMVL_, hinge point of the posterior MVL; (ii) ROI_TRIG_, intertrigonal mitral annulus; (iii) ROI_AMVL_, AMVL tip; and (iv) ROI_AAO_, anterior aortic annulus. The labelled dataset was then exported to MATLAB® (R2012b) where an in-house script based on a linear quadratic estimation (adaptation of the Kalman filtering technique) automatically target-tracked the dynamic excursion of each ROI across all frames of the dataset (*Figure 3*). The Kalman filtering technique based on linear quadratic estimation as applied to this study is a widely used motion tracking algorithm that has been vastly applied across domains from sensor-based systems to image processing. Its use has been validated in several biomedical settings previously.^17–19^ We adapted this previously validated algorithm to the motion tracking of aorto-mitral ROIs in CMR data. The algorithm operated recursively on the ROIs tracked over one R–R interval and handled background image noise by producing a statistically optimal estimate of the underlying kinetic state. Resultant aorto-mitral ROI paths were normalized and graphed to provide a visual representation relative to the cardiac cycle, and maximum antero-posterior and supero-inferior displacements per ROI were then calculated.

### Reproducibility

Intraobserver variability was obtained by comparing ROI path coordinates by the same blinded observer using 10 random cases with a measuring time interval in excess of 2 weeks. Interobserver variability was assessed by two experienced and double-blinded observers.

### Statistical analysis

Statistical analysis and data visualization were performed in the R programming language for statistical computing (version 4.1.2, The R Foundation for Statistical Computing). Descriptive data are expressed as mean ± SD except where otherwise stated. The distribution of data was assessed on histograms and using the Shapiro–Wilk test. Categorical baseline variables were compared using the *χ*^2^ or Fisher exact tests. Correlation between variables was assessed with Spearman’s rank-order correlation coefficient or the point–biserial correlation coefficient as appropriate. A two-way random, single-measures intraclass correlation coefficient (ICC) was used to compare the variability of motion track data within and between readers. The coefficient of variation (CoV) was measured as the ratio of the SD to the mean, thus summarizing the level of dispersion around the mean. To compare ROI displacement-vs.-time paths between participant groups, covariate models were constructed for the analysis of response *X*/*Y* to terms *T*|*A*, where *A* was the fixed factor (e.g. participant group) and covariate *T* was the (numeric) relative time along the cardiac cycle as a % of the R–R interval. A two-factor fully cross-factored analysis of variance model (*X*/*Y* = *T*|*A* + *e*, where epsilon signifies full replication) was used to determine the statistical significance of different ROI paths between groups. Two-sided values of *P* < 0.05 were considered significant.

## Results

The baseline clinical and demographic characteristics of study participants are summarized in *Table 1*. Cases and controls were matched in terms of age, sex, ethnicity, and BSA. As previously reported, there were no significant differences between G+LVH− and controls in terms of LV volumes, ejection fraction, wall thickness, mass, and LA size (all *P* > 0.05), but significant differences across these parameters were noted between G+LVH+ and controls. Compared with controls, G+LVH− had longer AMVL and narrower LVOT (*P* < 0.0001). The majority of G+LVH+ expressed sigmoid septal asymmetric septal hypertrophy (ASH).

**Table 1:** Demographic and clinical characteristics of study participants

Variable	G+LVH−	Controls	*P* ^a^	G+LVH+	Controls	*P* ^a^
	(*n* = 36)	(*n* = 36)		(*n* = 31)	(*n* = 31)	
Age (years)	31.3 ± 13.8	33.4 ± 12.2	0.05	47 ± 12	45 ± 14	0.67
Male/female	12/24	12/24	1.00	19/12	19/12	1.00
Ethnicity^b^	A = 35 C = 0 D = 1 E = 0	A = 35 C = 0 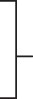 D = 1 E = 0	1.00	A = 21 C = 2 D = 5 E = 3	A = 21 C = 2 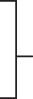 D = 5 E = 3	1.00
BSA (m^2^)	1.8 ± 0.2	1.8 ± 0.2	0.60	1.9 ± 0.2	1.9 ± 0.2	0.92
LVEDV*i* (mL/m^2^)	73 ± 10	74 ± 11	0.60	74 ± 22	72 ± 9	0.66
LVESV*i* (mL/m^2^)	21 ± 4	24 ± 6	0.05	22 ± 15	23 ± 5	0.78
LV EF (%)	71 ± 4	68 ± 4	**0.02**	71 ± 10	68 ± 5	0.13
LV mass*i* (g/m^2^)	58 ± 12	59 ± 13	0.65	117 ± 51	65 ± 14	**<0.0001**
SWTd (mm)	8.9 ± 1.9	8.4 ± 1.2	0.13	21.0 ± 6.5	9.0 ± 1.4	**<0.0001**
PWTd (mm)	6.4 ± 1.4	6.4 ± 1.4	0.86	9.7 ± 3.2	7.06 ± 1.6	**<0.001**
SdPdR	1.4 ± 0.3	1.4 ± 0.3	0.59	2.3 ± 1.0	1.3 ± 0.3	**<0.0001**
LA area*i* (cm^2^/m^2^)	10.9 ± 1.5	10.7 ± 1.4	0.73	15.3 ± 3.4	10.0 ± 1.2	**<0.0001**
AMVL (mm)	23.5 ± 3.0	19.9 ± 3.1	**<0.0001**	24.6 ± 4.6	19.6 ± 2.8	**<0.0001**
LVOTEDd (mm)	21.4 ± 2.4	21.5 ± 1.9	**<0.0001**	20.4 ± 3.0	21.8 ± 4.2	**<0.0001**
LVOTESd (mm)	17.6 ± 2.4	18.2 ± 2.2	**<0.0001**	16.7 ± 3.2	19.2 ± 2.1	**<0.0001**
LVOTO^c^						
Present Absent	2 (5.5%) 32 (88.9%)	0 36 (100%) 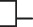	**0.04**	17 (54.9%) 14 (45.1%)	0 31 (100%) 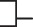	**<0.0001**
Resting SAM						
None Partial Complete Chordal	34 (94.4%) 2 (5.6%) 0 0	36 0 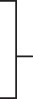 0 0	0.15	14 (45.1%) 6 (19.4%) 11 (35.5%) 0	31 0 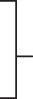 0 0	**<0.0001**
LVH distribution						
None Sigmoid septal ASH Reversed septal contour ASH Mid-ventricular LVH Apical HCM Symmetric HCM	36 0 0 0 0 0	36 0 0 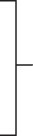 0 0 0	1.00	0 26 0 2 1 2	31 0 0 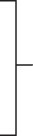 0 0 0	**<0.0001**
LVOT–basal septal angle (**°**)	143.2 ± 8.6	142.9 ± 7.9	0.911	138.3 ± 12.1	137.8 ± 12.3	0.805
LGE volume (mL)	49.3 ± 17.6	/	/	112.9 ± 55.7	/	/
Normalized max. antero-posterior displacement						
ROI_PMVL_	51.7 ± 1.4	51.1 ± 2.6	**0.048**	41.1 ± 6.6	42.2 ± 7.3	0.437
ROI_TRIG_	16.4 ± 2.4	15.4 ± 3.3	0.161	91.1 ± 3.7	90.6 ± 4.8	**0.049**
ROI_AMVL_	19.1 ± 8.6	17.7 ± 9.2	**<0.0001**	39.4 ± 3.2	38.8 ± 3.2	**<0.0001**
ROI_AAO_	71.8 ± 2.1	69.8 ± 3.0	**0.014**	99.0 ± 6.3	100.5 ± 7.3	**0.008**
Normalized max. supero-inferior displacement						
ROI_PMVL_	61.1 ± 3.2	61.8 ± 3.6	0.888	55.1 ± 4.2	57.7 ± 4.2	0.131
ROI_TRIG_	26.4 ± 2.7	24.9 ± 3.4	**0.012**	98.8 ± 2.4	101.2 ± 4.4	**0.007**
ROI_AMVL_	23.4 ± 9.7	23.9 ± 9.7	0.806	47.7 ± 1.2	50.7 ± 3.1	**<0.0001**
ROI_AAO_	79.1 ± 1.9	78.2 ± 3.4	0.054	107.5 ± 4.0	110.7 ± 6.6	**0.042**

All data are expressed as mean ± SD or counts as appropriate.

AMVL, anterior mitral valve leaflet; ASH, asymmetric septal hypertrophy; BSA, body surface area; EF, ejection fraction; G+/G−, sarcomere gene mutation positive/negative; LA area*i*, left atrial area indexed to BSA; LVEDV*i*, left ventricular end-diastolic volume indexed to BSA; LVESV*i*, left ventricular end-systolic volume indexed to BSA; LVH+/LVH−, left ventricular hypertrophy present/absent; LVOTED/Sd, left ventricular end-diastolic/systolic diameter; LVOTO, left ventricular outflow tract obstruction; LV mass*i*, left ventricular mass indexed to BSA; Max., maximum; MVAd, mitral valve annular diameter (in end-diastole); SAM, systolic anterior motion; SdPdR, diastolic septal to diastolic posterior wall thickness ratio; S/PWTd, maximal septal/posterior wall thickness in diastole.

^a^Significant *P* values are highlighted in bold.

^b^Ethnic headings are defined in accordance with UK Office for National Statistics guidance on national standards: A = White; B = Mixed; C = Asian or Asian Black; D = Black or Black British; E = Chinese or other ethnic groups (including Arab).

^c^LVOTO assessed by echocardiography.

### Kinetics of the aorto-mitral apparatus

**Figure 4:**
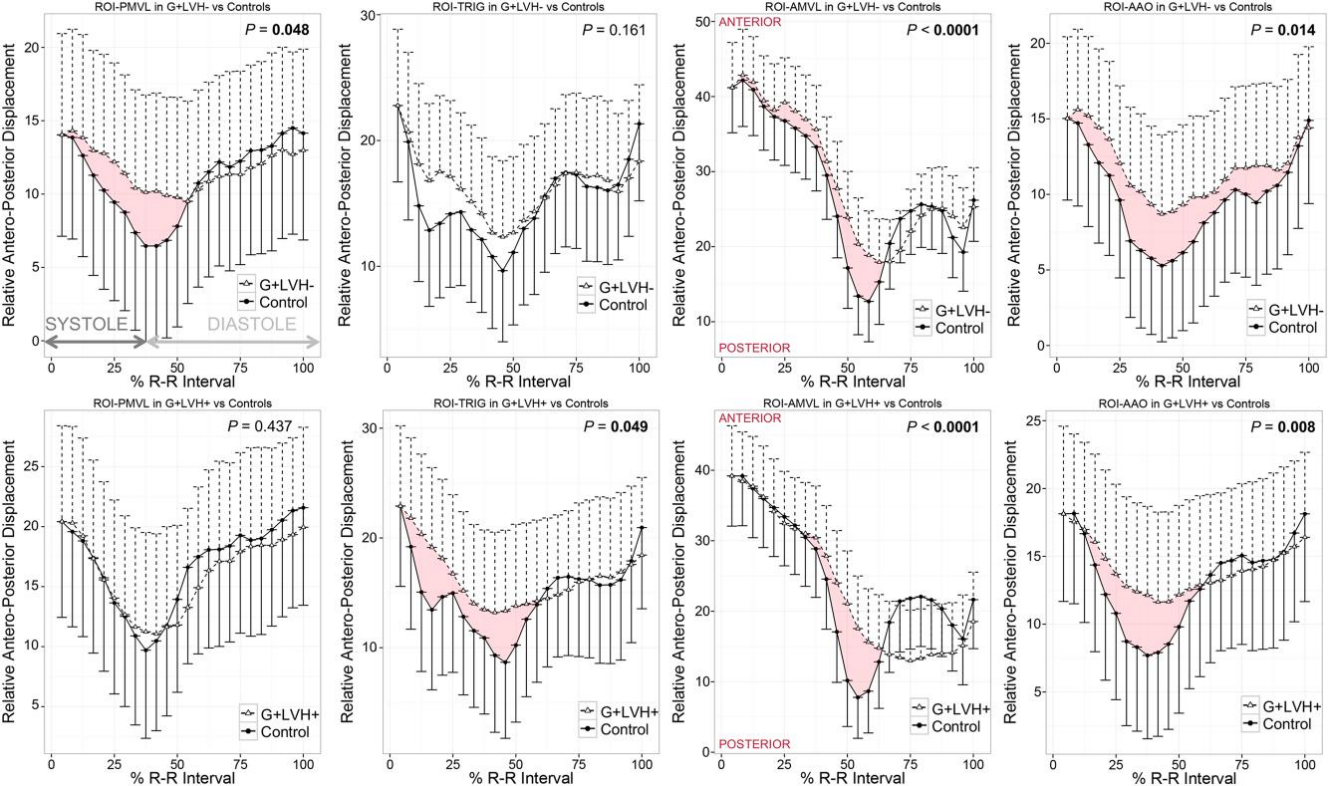
Antero-posterior kinetics of the aorto-mitral annulus in G+LVH− and G+LVH+ compared with controls. Two-dimensional antero-posterior displacement-vs.-time plots in G+LVH−/G+LVH+ vs. matched controls normalized for R–R intervals and baseline positions. Motion tracking software measured the ROI position in each cine frame during the cardiac cycle. Data symbols indicate mean cohort values, and unidirectional whiskers indicate standard errors. Significant *P* values are highlighted in bold. Red ribbons highlight statistically significant SAM of the AMVL in overt and subclinical HCM. HCM, hypertrophic cardiomyopathy; SAM, systolic anterior motion. Abbreviations as in *Figures 1* and *2*.

**Figure 5:**
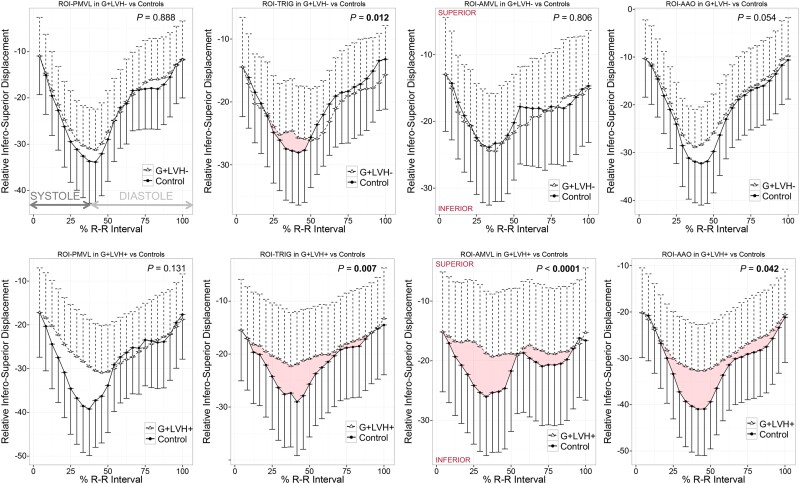
Infero-superior kinetics of the aorto-mitral annulus in G+LVH− and G+LVH+ compared with controls. Two-dimensional infero-superior displacement-vs.-time plots in G+LVH−/G+LVH+ vs. matched controls normalized for R–R intervals and baseline positions. Data symbols indicate mean cohort values, and unidirectional whiskers indicate standard errors. Significant *P* values are highlighted in bold. Abbreviations as in *Figures 1* and *2*.

Compared with controls, normalized two-dimensional displacement-vs.-time plots in G+LVH− revealed subtle but significant SAM of the AMVL (measurable as an antero-posterior displacement, *P* < 0.0001, *Figure 4*) and reduced longitudinal excursion of both ROI_AAO_ (*P* = 0.014) and ROI_PMVL_ (*P* = 0.048, *Figure 4*). As expected, AMVL SAM was obvious in G+LVH+ compared with controls (*P* < 0.0001, *Figure 5*) together with reduced longitudinal excursion of ROI_TRIG_ (*P* = 0.049) and ROI_AAO_ (*P* = 0.008).

In G+LVH−, most of the aorto-mitral mechanical differences compared with controls occurred in the antero-posterior direction, while in G+LVH+ patients, abnormal aorto-mitral kinetics were more pervasive, occurring in both antero-posterior and infero-superior directions.

### Relationship of aorto-mitral kinetics with other atrioventricular structures

In G+LVH−, there was a significant positive correlation between aorto-mitral ROI displacements and maximal end-diastolic septal wall thickness (*r*_s_ ranging from 0.17 to 0.31, *P* < 0.03) and LVOT end-diastolic diameter (*r*_s_ ranging from 0.25 to 0.26, *P* < 0.04 both). There was an inverse correlation between aorto-mitral ROI displacements and AMVL lengths (*r*_s_ ranging from −0.33 to −0.23, *P* < 0.02) and basal septal angles (*r*_s_ ranging from −0.49 to −0.38, *P* < 0.001). In G+LVH+, there was a significant negative correlation between aorto-mitral ROI displacements and basal septal angles (*r*_s_ ranging from −0.51 to −0.24, *P* < 0.04).

### Relationship of aorto-mitral kinetics with the focal fibrosis burden

Across all HCM patients (subclinical and overt), there was a significant positive correlation between the global LV burden of LGE and the displacement of ROI_PMVL_ (*r*_s_ 0.48, *P* = 0.028); between LGE volumes in Segment 8 (mid-anteroseptum) and displacements of ROI_TRIG_ and ROI_AMVL_ (*r*_s_ 0.52, *P* = 0.016 and *r*_s_ 0.52, *P* = 0.015, respectively); and between the LGE burden in mid-LV segments and the ROI_PMVL_ and ROI_AMVL_ (*r*_s_ 0.53, *P* = 0.013 and *r*_s_ 0.50, *P* = 0.022, respectively).

### Intra- and interobserver variability

ICCs for repeated intra- and interobserver measurements of ROI *X*/*Y* coordinates were high [ICC, 0.94 (95% confidence interval: 0.88–0.97) and 0.92 (95% confidence interval: 0.86–0.95), respectively]. CoVs for intra- and interobserver readings were <3% both.

## Discussion

Two key findings from our study (*Graphical Abstract*) are as follows: (i) MV abnormalities in HCM go beyond mere elongation of the anterior and posterior MVL, with abnormal motion affecting the ‘entire’ aorto-mitral apparatus; (ii) a degree of AMVL SAM precedes the development of LVH in HCM sarcomere gene mutation carriers.

That AMVL SAM leads to pathological MR and LVOTO is well known in HCM patients with established LVH, but we show how SAM of the abnormally elongated AMVL is already occurring in sarcomere gene mutation carriers well before the development of LVH.

We expand on the knowledge base established in prior studies investigating the motion of the aorto-mitral apparatus in subclinical and overt HCM. Groarke *et al.*^20^ previously demonstrated the presence of MVL SAM in both subclinical and overt HCM patients. The present study confirms this finding with the employment of more comprehensive valve motion tracking from tip to base of the anterior and posterior MVLs, in addition to the use of cine CMR and LGE analysis. By two-dimensional echocardiography, Groarke *et al.* found no significant difference in absolute AMVL lengths between subclinical HCM and controls, but after indexation to LV cavity size, AMVL elongation in subclinical HCM was significant. Other studies, particularly those using higher-resolution CMR imaging, have consistently measured significantly longer absolute AMVL lengths in subclinical HCM patients compared with controls.^1,2,21^ Indeed, AMVL elongation is now widely accepted to be one of the phenotypic traits of the subclinical HCM phenotype by CMR.^8^

Various theories have been proposed to elucidate the putative mechanisms of SAM in HCM. The Venturi theory was previously widely accepted and describes an effect caused by raised flow velocities in an LVOT geometry altered by septal hypertrophy, which pulls the elongated leaflets into the LVOT causing obstruction.^22^ However, this theory fails to explain numerous features of SAM; importantly, it begins even before ventricular ejection commences in patients with HCM.^3^ Vector flow mapping has since been used to show that drag forces are the predominant mechanical force producing this excursion. Indeed, it has been shown that MVLs are pushed anteriorly by a posterior vortex created by late diastolic mitral inflow, now known as diastolic anterior motion of the MV.^23^

The precise mechanism for AMVL elongation in HCM is incompletely understood. Hypotheses proposing abnormal valvulogenesis *in utero*,^24^ or that AMVL elongation occurs as a compensatory response to the increased endothelial shear rate in the narrowed LVOT,^25^ insufficiently explain this abnormality. Indeed, within this study, we detected AMVL elongation even in those without LVOT narrowing. Previous work has posited that increased myocardial trabecular complexity may increment the mass of secretogenic myocardial tissue,^8^ thereby driving the aberrant trajectory of valvulogenesis. Present findings point towards a multifaceted aetiology of the phenomenon. HCM sarcomere gene mutation carriers may be more likely to experience post-natal MVL stretch due to a combination of evolving LVOT abnormalities and intrinsic MVL aberrations. The strength of these conclusions is however limited by the cross-sectional nature of our data. Further longitudinal follow-up studies of G+LVH− patients from childhood to late adulthood are needed to enhance our understanding of MVL changes over time and their temporal relationship to overall phenotype transition.

The two MVLs normally appose at a solitary zone laterally and at the ROI_TRIG_, which has a contractile role, assisting papillary muscles and tendinous cords to regulate MV function.^26^ Speckle tracking CMR has shown that in HCM with asymmetric LVH, hypertrophied as well as non-hypertrophied myocardial segments exhibit reduced radial, circumferential, and longitudinal peak strain and that non-hypertrophied segments additionally showed reduced peak displacement.^27^ It is highly likely that the regional wall deformation and dysfunction resulting from myocardial fibrosis and disarray in the three myocardial fibre layers extend to the mitral annulus in HCM, producing the abnormal motion of the aorto-mitral apparatus we have observed. Reduced longitudinal excursion of the aorto-mitral apparatus was most prominent at the ROI_PMVL_ during systole, correlating with the LGE burden, but was noted throughout the cardiac cycle at ROI_AAO_. Abnormalities in geometric dynamics of the mitral apparatus have previously been demonstrated in the echocardiographic assessment of patients with HCM. Hwang *et al.*^28^ concluded that displacement of the MV coaptation point towards the septum affected the degree of LVOTO as the residual MVL is displaced into the LV outflow stream. This effect is further attenuated by the abnormal proximity of the papillary muscles. Anomalous papillary muscles have been previously described in HCM. In a CMR study, Kwon *et al.*^29^ observed anterior displacement of the anterolateral papillary muscle in the majority of patients with HCM, as well as a commonly present bifid papillary muscle. This anomalous papillary muscle displacement, along with the abnormal septal convexity of the LV septum in subclinical HCM,^9^ further shifts the MV into an anterior position that produces an overlap between the inflow and outflow portions of the LV by moving the MVLs closer to the ventricular septum and the LVOT. This results in laxity of the valvular apparatus, making the AMVL more susceptible to excursion into the LVOT by associated drag forces. It is possible that the reduction in longitudinal excursion of the aorto-mitral apparatus may further increment the sub-mitral abnormalities present in HCM, augmenting the degree of LVOTO.

Based on our data, it is plausible that over time, repetitive and abnormal kinesis of the aorto-mitral apparatus mechanically stretches the MVLs and encourages SAM that eventually leads to the familiar narrowing of the LVOT. Furthermore, abnormal aorto-mitral dynamics will result in MR from the loss of the systolic zone of leaflet apposition. Sustained abnormal aorto-mitral kinetics during the lifespan of HCM patients predispose to contraction band necrosis^30^ at the annular region and its surrounding tissues. Indeed, we found a significant positive correlation between aorto-mitral kinetics and the global LV burden of LGE, particularly in the mid-anteroseptum and all other mid-LV segments. An appreciation of the potential early role of the aorto-mitral annulus and AMVL in morbidity causation in HCM has the potential to inspire new and earlier management approaches. The development of disease-modifying therapies for subclinical HCM has been slow due to the limited understanding of the pathways leading from sarcomere gene mutation carriage to overt disease, but progress is being made. Early animal studies using diltiazem^31^ showed promise at attenuating disease emergence in HCM mutation–positive mice, and a subsequent randomized trial of the drug in G+LVH− humans also demonstrated favourable outcomes. Similarly, mavacamten, a novel cardiac myosin ATPase inhibitor, may prevent the development of myocardial fibrosis and cardiomyocyte disarray.^32^ At present, the efficacy of mavacamten has only been demonstrated in patients with symptomatic HCM,^33^ and its role in subclinical HCM, and effect on the aorto-mitral apparatus, remains unclear. Alternative medical therapy may delay the accrual of myocardial fibrosis and thus preserve normal aorto-mitral behaviour. Regular administration of N-acetylcysteine may have a beneficial effect on myocardial fibrosis,^34^ and inhibition of the renin–angiotensin–aldosterone system may reduce the extent of LGE.^35^ While these results were not replicated in larger studies in established, obstructive HCM,^36^ the effects in LVH− patients remain unclear and early initiation of therapy may prevent fibrosis and subsequent aorto-mitral dysfunction.

Atrioventricular junction tracking tools, such as the one we describe, are not new to CMR^37,38^ and have been successfully used to measure diastolic function in healthy hearts as well as in overt HCM. One published feature tracking tool reconstructs the three-dimensional mitral annular sweep volume using six ROIs implanted onto the three long-axis cines.^37^ However, this method is limited to the mitral annular plane and does not capture motion of the ROI_AMVL_, where we found most of the abnormalities. Furthermore, the ROI pairs were tracked across three different long-axis cines, each acquired at non-identical time points, with the potential for inter-heartbeat differences and altered respiratory variation. Our method has the advantage of assessing all four aorto-mitral ROIs in a single standard three-chamber view acquired as part of every routine clinical CMR examination, using the standard cine SSFP sequence without the need for additional imaging pulse sequences and associated acquisition time.

Limitations of this study are that it is single-centre and cross-sectional in design.^8^ While findings from this adult cohort provide some insights into the kinetics of the aorto-mitral apparatus in HCM, further work is needed, particularly to include paediatric HCM. The aorto-mitral tracking tool is sensitive to respiratory motion and gating artefacts and image rotation and is dependent on high-quality three-chamber cines for optimal performance.

## Conclusion

Dyskinesia of the aorto-mitral apparatus, including SAM of the AMVL, is already noticeable in subclinical HCM before the development of LVH or LA enlargement. These data have the potential to improve our understanding of early phenotype development and LVOTO predisposition in HCM.

## Data Availability

The data underlying this article are available via our institutional GitHub account.
